# Application of quantitative real-time PCR to detect *Mink Circovirus* in minks, foxes and raccoon dogs in northern China

**DOI:** 10.3389/fmicb.2023.1205297

**Published:** 2023-07-31

**Authors:** Yingyu Liu, Chenyan Sheng, Yu Zhou, Jianming Li, Qinglong Gong, Kun Shi, Fei Liu, Lihui Xu, Zhenzhen Cui, Xue Leng, Rui Du

**Affiliations:** ^1^College of Animal Science and Technology, Jilin Agricultural University, Changchun, China; ^2^Huizhou Customs District P.R. China, Huizhou, China; ^3^College of Chinese Medicine Materials, Jilin Agricultural University, Changchun, China

**Keywords:** *Mink Circovirus*, quantitative real-time PCR, epidemiological investigation, detection, fur-bearing animals

## Abstract

Mink circovirus disease caused by *Mink Circovirus* (MiCV) is a serious infectious disease of mink that has become prevalent in recent years in China, severely affecting the reproductive performance of mink and causing significant economic losses to farms. To date, there have been few studies on MiCV, its pathogenic mechanism is not clear, and there is no effective vaccine or drug to prevent and control the disease. Therefore, it is necessary to establish a rapid and reliable molecular diagnostic method, which would aid future studies of this novel virus. In our study, we developed a sensitive and specific TaqMan-based quantitative real-time PCR assay targeting the MiCV Cap gene. The assay showed no cross-reaction with other tested animal viruses. The assay is highly sensitive, with a detection limit of as low as 10 plasmid DNA copies and 2.38 × 10^−2^ pg of viral DNA. The intra and inter--assay coefficients of variation were both low. The positive detection rate of MiCV in clinical samples from minks, foxes, and raccoon dogs were 58.8% (133/226), 50.7% (72/142), and 42.2% (54/128), respectively, giving a total positive detection rate of 52.2% (259/496). Higher contamination levels were observed in samples from the environment in direct or indirect contact with animals, with a total positive detection rate of 75.1% (220/293). These epidemiological results showed that minks, foxes, and raccoon dogs had high infection rates of MiCV. This was also the first study to detect MiCV on the ground and equipment of fur-bearing animal farms. Our assay is highly sensitive and specific for the diagnosis and quantification of MiCV, and should provide a reliable real-time tool for epidemiological and pathogenetic study of MiCV infection.

## 1. Introduction

*Circoviruses* have various hosts among mammals and birds (Li et al., 2010; Decaro et al., 2014). In recent years, in addition to the well-known *Porcine Circovirus*, *Circoviruses* that can infect canines, penguins, bats, and other animals have been reported (Piewbang et al., 2018; Matsumoto et al., 2019; Morandini et al., 2019; Canuti et al., 2022). Mink circovirus disease, caused by a new *Circovirus* member, *Mink Circovirus* (MiCV), has only been discovered in China and is reported to be able to infect foxes and raccoon dogs (Yang et al., 2018). Taxonomically, MiCV belongs to the genus *Circovirus* in the family *Circoviridae*. *Circoviruses* are small envelope-free icosahedral viruses, with a diameter of 15–25 nm, and a circular single-stranded DNA genome, which is the smallest known auto replicating viral genome (Breitbart et al., 2017; Rosario et al., 2017). The genome of MiCV comprises about 1700 nucleotides, including two main open reading frames (ORFs), one encoding the replication-related protein (Rep) and the other encoding viral capsid protein (Cap; Ge et al., 2018a). In *Circovirus*, the ORF encoding Rep is on the sense strand of the genome, while the Rep of *Cyclovirus* is encoded by the complementary strand of double-stranded DNA (Breitbart et al., 2017; Rosario et al., 2017; Shulman and Davidson, 2017; Zhao et al., 2019). The Rep protein (35.8 kDa) is mainly involved in virus replication and plays an important role in virus proliferation, while Cap, is the main structural protein (27.8 kDa) and has good immunogenicity (Rosario et al., 2017).

Mink circovirus disease has a wide epidemic range in China. Existing epidemiological research results showed that the positive rate of MiCV was 30.30% in Heilongjiang Province, 38.46% in Jilin Province, 52.88% in Shandong Province, 58.46% in Liaoning Province and 67.90% in Hebei Province (Ge et al., 2018a). MiCV has caused great economic losses to the mink breeding industry. Minks infected with the virus showed poor appetite, mental fatigue, rough fur, diarrhea, and even death, and the virus easily causes mixed infection with Aleutian mink disease virus, Canine distemper virus and other viruses (Ge et al., 2018b). Most minks recovered spontaneously, however, their growth was poor, their fur quality was significantly reduced, and the reproductive capacity of females was lower than before infection. MiCV can be transmitted between minks of different ages.

In many places in China, minks, foxes, and raccoon dogs are kept on the same fur-bearing animal farms. A study showed that MiCV can infect not only minks, but also foxes and raccoon dogs，accompanied by similar clinical symptoms such as drowsiness, anorexia, pale mouth and unkempt fur (Yang et al., 2018). Besides, the persistence of the virus in the environment is also an important factor in the spread of the disease. To date, no large-scale MiCV prevalence studies in minks, foxes and raccoon dogs have been carried out. Viral contamination in the environment is also unknown. There have been few studies on MiCV, its pathogenic mechanism is not clear, and there is no effective vaccine and drug to treat the disease. Therefore, it is important to establish a rapid and effective detection method for disease prevention and control. Quantitative real-time PCR (qPCR) detection has the advantages of strong specificity, high sensitivity and relatively low cost (Abdel-Moneim et al., 2017; Tong et al., 2020; de Oliveira Lopes et al., 2021; Gong et al., 2021). It can quickly determine viruses in different tissue samples and has become an important tool in veterinary virology and disease control (Varga and James, 2006; Vázquez et al., 2017; Cui et al., 2018).

In this study, a TaqMan-based qPCR method was established to detect MiCV DNA. Subsequently, we detected MiCV DNA in clinical samples from minks, foxes, raccoon dogs and farm environments in China.

## 2. Materials and methods

### 2.1. Viruses and samples

MiCV strain HB3 (GenBank accession No. MK561562.1), Aleutian mink disease virus (AMDV) DL125 strain (Isolated by Dr. Liu of the Economic Animal Infectious Diseases Laboratory of Jilin Agricultural University in 2015 citation), Pseudorabies virus (PRV) JL03 strain, and Porcine circovirus 2 (PCV-2) were stored in the Economic Animal Infectious Diseases Laboratory of Jilin Agricultural University. The vaccine strains mink enteritis virus (MEV), canine distemper virus (CDV), and canine adenovirus type 2 (CAdV-2) were purchased from Teyan Biotechnology, Ltd., (Jilin, China) and QiLu Animal Health Products, Ltd., (Shandong, China), respectively.

Two hundred and twenty-six mink anal swab samples, 142 fox anal swab samples, 128 raccoon dog anal swab samples, and 293 environmental swab samples were collected from 6 farms in Jilin, Heilongjiang, and Shandong provinces in China from 2021 to 2022.

### 2.2. Primer design and synthesis

According to the MiCV genome sequences obtained in the laboratory in recent years and all 13 of MiCV genome sequences deposited in GenBank, DNAMAN (LynnonBiosoft, USA) was used to perform multiple comparisons to obtain a highly conserved region in the Cap region of the genome. Primer 5.0 (PREMIER Biosoft International, Palo Alto, California, USA) was used to design the required primers (real-time quantitative PCR primers and common PCR primers) and probes (Table 1). The primers and probes were synthesized by Comate Bioscience Co. Ltd., (Changchun, China).

**Table 1:** Primers and probes used in this study.

Name	Sequence (5′ to 3′)	Length (bp)	Position^a^
MiCV-F	AGGGCCTTTGGGCATCATTG	107	1,473-1,579
MiCV-R	CCCGCCTGCAAACTGAAGAA		
MiCV-Cap-F	TTAAGTTTGCTTTGGGAAATTGACT	684	1,020–1703
MiCV-Cap-R	ATGCCCGTAAGATCGCGATACTCGC		
MiCV-Probe	FAM-ACGGAGTTGCTGCAGATGCCACGGT-TAMR	-	1,529–1,553

a Nucleotide positions are designated according to the gene of MiCV strain HB3 (GenBank accession No. MK561562.1).

### 2.3. DNA extraction

All swab samples were repeatedly frozen and thawed three times and centrifuged. According to the manufacturer’s protocol, a Viral DNA Kit (Omega Biotek, Winooski, VT, USA) was used to extract DNA from 200 μl of the supernatant. After the DNA samples were eluted into 100 μl of Elution Buffer, they were stored at – 20°C until required.

### 2.4. Preparation of the standard plasmid

The 684 bp fragment of the Cap gene was amplified from the DNA of MiCV strain HB3 using PCR with the MiCV-Cap-F and MiCV-Cap-R primers. A Midi Purification Kit (Tiangen Biotech, Beijing, China) was used to purify the amplification products from the agarose gel, which were then ligated into the vector pMD18-T (Takara Biotechnology, Dalian, China) to construct recombinant plasmid pMD-MiCV. pMD-MiCV was transformed into *Escherichia coli* DH5α cells (TransGen Biotech, Beijing, China), purified. Using a plasmid miniprep Kit (Axygen A Corning Brand, Suzhou, China), and verified by sequencing (carried out by Comate Bioscience, Changchun, China). The target plasmid pMD-MiCV had an original concentration of 210.47 ng/μl, as measured using a Nanodrop One spectrophotometer (Thermo Scientific, Wilmington, DE, USA). Ten-fold serial dilutions of plasmid DNA in elution buffer were created and stored at −20°C until further testing. According to the following formula: the copy number of the plasmid (copies/μl) = [concentration of plasmid (ng/μl) × (6.02 × 10^23^)]/[(plasmid length × 660 × 10^9^)], the copy number of the plasmid was 5.68 × 10^10^ copies/μl. The original plasmid was diluted using elution buffer to 1.0 × 10^10^ copies/μl, stored as the standard plasmid-20°C until required.

### 2.5. Establishment of a standard curve for qPCR

The standard plasmid pMD-MiCV was serially diluted 10 times with elution buffer, 1.0 × 10^6^ copies/μl to 1.0 × 10^1^ copies/μl as templates to establish the standard curve, created by plotting the logarithm of the plasmid copy number against the measured cycle threshold (Ct) values. The standard curve, the correlation coefficient of the standard curve, and the qPCR efficiencies were calculated using the GraphPad Prism 7 software (GraphPad Inc., La Jolla, CA, USA). The amount of DNA quantified in each sample was expressed as number of copies per reaction. The final volume was 20 μl including 10 μl of TB Green® Premix Ex Taq™ II (Takara Biomedical Technology, Beijing, China), 0.8 μl each of the MiCV-F and MiCV-R primers (10 μmol/L), 0.4 μl of the probe MiCV-P (10 μmol/L), 1 μl of template, and 6 μl of ddH_2_O. The qPCR reaction was carried out in eight tubes by fluorescence quantitative PCR in qTOWER3 G instrument (Analytik Jena AG, Jena, Germany). The procedure comprised incubation at 95°C for 3 min, followed by 45 cycles of 95°C for 10 s and 57°C for 40 s.

### 2.6. Specificity, sensitivity, and repeatability analysis

To exclude qPCR cross-reactivities between MiCV and other pathogens, six viruses, namely, AMDV, PRV (PRV has occurred in mink in recent years, and might be related to eating pigs’ viscera), PCV-2, MEV, CDV and CAdV-2 were subjected to qPCR to confirm the specificity of the technique. MiCV strain HB3 was used as the positive control and ddH_2_O was used as the negative control, and each sample was repeated 3 times.

To compare the sensitivity of qPCR and conventional PCR, dilutions of plasmid pMD-MiCV (1.0 × 10^5^ copies/μl to1.0 × 10^0^ copies/μl) and DNA of MiCV strain HB3 (2.38 × 10^2^ pg/μl to 2.38 × 10^−3^ pg/μl) were used as templates for qPCR and conventional PCR. The conventional PCR reaction was performed using the primer pair MiCV-cap-F and MiCV-cap-R. Besides, the diagnostic sensitivity of the qPCR was tested by the positive clinical samples verified by sequencing.

Three different dilutions of standard plasmid (1.0 × 10^2^ copies/μl, 1.0 × 10^4^ copies/μl, and 1.0 × 10^6^ copies/μl) were used for amplification in three parallel assays under the same conditions. The coefficient of variation (CV) was calculated according to the formula CV = (SD [Ct value]/overall average [Ct value]) × 100, to evaluate the intra-and inter-assay repeatability and stability in the qPCR method.

### 2.7. Clinical samples

A total of 789 clinical samples were collected from different fur-bearing animal farms in China. The samples included anal swab samples and environmental swab samples. The anal swab samples were collected by inserting sterile cotton swab into the anus about 2 to 3 cm, gently rotating it, and pulling it out. The anal swab samples were then placed into sampling tubes that contained 2 ml of sterile phosphate-buffered saline. The environmental swab samples were collected using a sterile cotton swab to clean each sampling area including cages, troughs, soil under the cages, sewage under the cages, aisle floors, breeder’s clothes, and equipment, for 20 to 30 s, then placing it into the sample tubes holding 2 ml of sterile phosphate-buffered saline. All samples were stored at −80°C immediately after being transported back to the laboratory at low temperature. The qPCR assays were performed on these samples for MiCV detection.

## 3. Results

### 3.1. Establishment of the standard curve for qPCR

**Figure 1:**
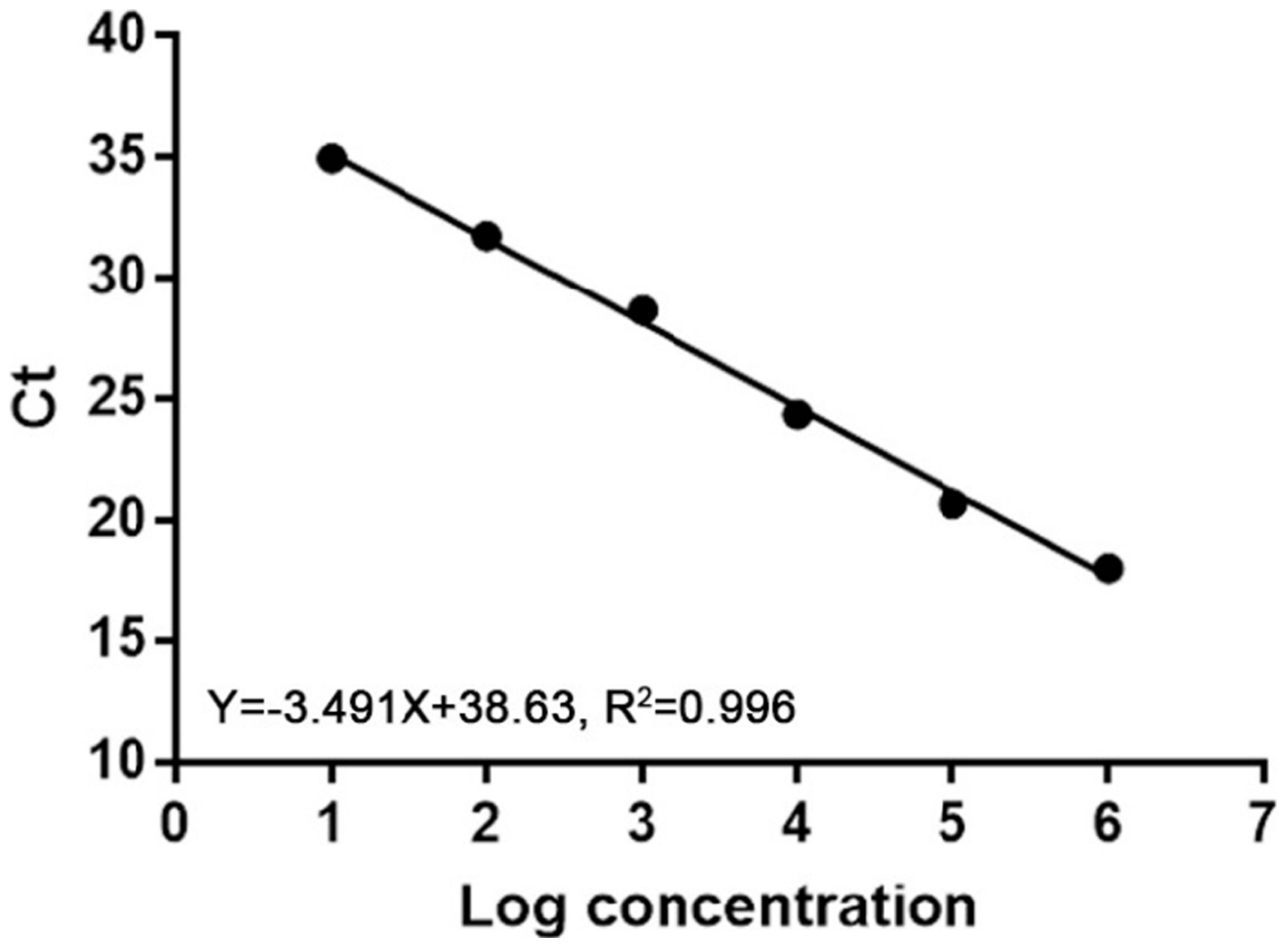
Standard curve of real-time PCR to detect MiCV. The correlation coefficient (R^2^) was 0.996 and the slope was −3.491.

Ten-fold serial dilutions of plasmids were used to construct a standard curve by plotting the logarithm of the plasmid copy number against the measured Ct values (Figure 1). The standard curve had a wide dynamic range of 10^1^ to10^6^ copies/μl with a linear correlation (R^2^) of 0.996 between the Ct value and the logarithm of the plasmid copy number. The amplification efficiency of the obtained qPCR was 93%.

### 3.2. Specificity, sensitivity, and repeatability of the qPCR

**Figure 2:**
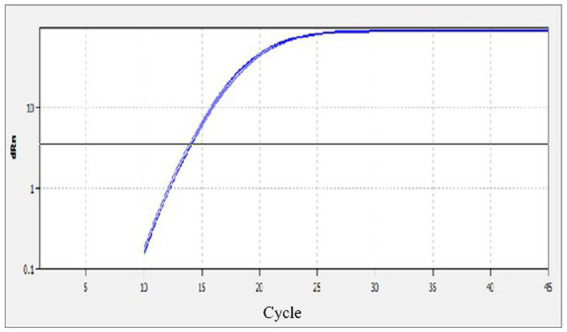
Specificity of the qPCR assay. The templates included DNA or cDNA of MiCV, AMDV, PRV, PCV-2, MEV, CDV, CAdV2 and the water control.

The specificity of the qPCR assay was evaluated using eight different reactions, which included, MiCV, AMDV, PRV, PCV-2, MEV, CDV, CAdV2 and a negative water control. Strong fluorescent signals were obtained from reactions with MiCV in three replications; while no signals were obtained from the other six virus samples and the water control (Figure 2). The results demonstrated that the MiCV assay specifically detected the target virus without cross detection of any non-target pathogens.

**Figure 3:**
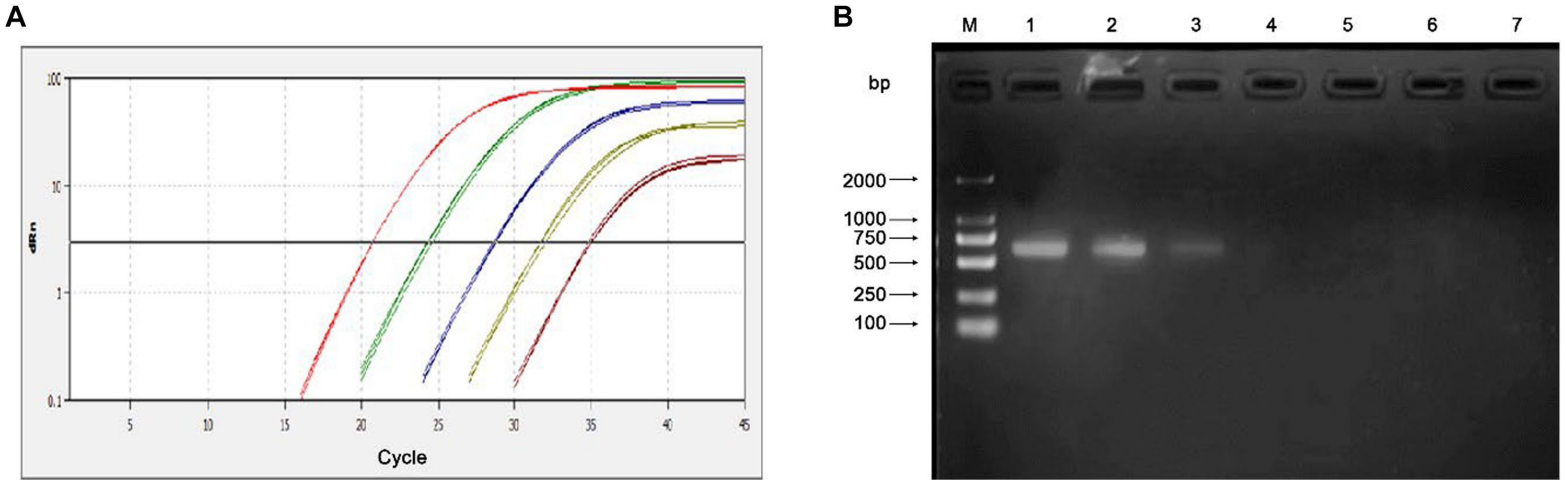
Sensitivity of the qPCR assay for the plasmid DNA of pMD-MiCV. **(A)** The qPCR amplification curve. **(B)** Electrophoresis of conventional PCR reactions. The template concentrations of plasmid DNA ranged from 1.0 × 10^5^ copies/μl to 1.0 × 10^0^ copies/μl. A negative control was included.

**Figure 4:**
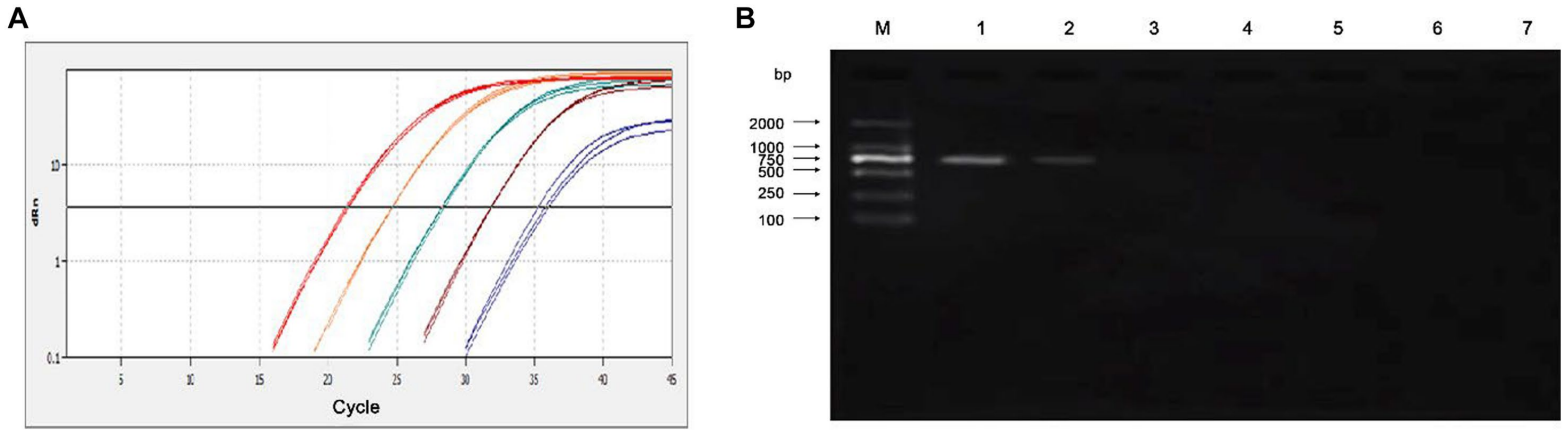
Sensitivity of the qPCR assay for the virus DNA of MiCV strain HB3. **(A)** The qPCR amplification curve. **(B)** Electrophoresis of conventional PCR reactions. The template concentrations of virus DNA ranged from 2.38 × 10^2^ pg/μl to 2.38 × 10^−3^ pg/μl. A negative control was included.

The sensitivity of the qPCR assays were evaluated by testing 10-fold serial dilutions of the DNA standards and the positive clinical samples. For the standard plasmid DNA of pMD-MiCV, the detection limit of qPCR was 1.0 × 10^1^ copies/μl, while the detection limit of conventional PCR was 1.0 × 10^3^ copies/μl (Figures 3A,B). For the virus DNA of MiCV strain HB3, the detection limit of qPCR was 2.38 × 10^−2^ pg/μl, while the detection limit of conventional PCR was 2.38 × 10^1^ pg/μl (Figures 4A,B). For the positive clinical samples, 35 of 35 positive samples were verified as positive by qPCR assay. Thus, the diagnostic sensitivity of the qPCR was 100%.

The intra-and inter-assay reproducibility was assessed using three dilutions of the standard plasmid pMD-MiCV DNA (1.0 × 10^2^ copies/μl, 1.0 × 10^4^ copies/μl, and 1.0 × 10^6^ copies/μl). The values of the intra-assay co-efficient of variation (CV) ranged from 0.46 to 0.96%, and the values of the inter-assay CV ranged from 1.37 to 1.98%, indicating that the qPCR method was highly reproducible.

### 3.3. Detection of the clinical samples

The application of qPCR was evaluated by detecting 226 mink anal swabs, 142 fox anal swabs and 128 raccoon dog anal swabs. The positive detection rates of MiCV in minks, foxes and raccoon dogs were 58.8, 50.7, and 42.2%, respectively (Table 2). The total positive detection rate was 52.2%. The positive detection rate of MiCV in minks was higher than that in foxes and raccoon dogs. The positive detection rate of MiCV in fur-bearing animals ≥1 year old was 55.1%, and was 49.6% in fur-bearing animals <1 year old. The positive detection rate in male fur-bearing animals was 51.0%, and was 53.5% in female fur-bearing animals (Table 3). Environmental samples from fur-bearing animal farms had a higher positive detection rate of 75.1%. Among them, the positive rates were higher in samples from cages, troughs, soil under the cages and sewage under the cages than in samples from floors, breeder’s clothes, and equipment (Table 4).

**Table 2:** The prevalence of MiCV in the clinical samples used in this study.

Province		Mink			Fox			Raccoon dog	
	Number	Positive number	Positive rate (%)	Number	Positive number	Positive rate (%)	Number	Positive number	Positive rate (%)
Heilongjiang	75	42	56.0	42	22	52.4	41	18	43.9
Jilin	83	46	55.4	55	28	50.9	48	19	39.6
Shandong	68	45	66.2	45	22	48.9	39	17	43.6
Total	226	133	58.8	142	72	50.7	128	54	42.2

**Table 3:** MiCV positivity in fur-bearing animals of different ages and sexes.

Groups		Number	Positive number	Positive rate (%)
Ages	≥1 year old	234	129	55.1
	<1 year old	262	130	49.6
	Total	496	259	52.2
Gender	Male	251	128	51.0
	Female	245	131	53.5
	Total	496	259	52.2

**Table 4:** Detection rate of MiCV in the environment of fur-bearing animal farms.

Source	Number	Positive number	Positive rate (%)
Cages	68	55	80.9
Troughs	53	44	83.0
Soil under the cages	56	43	76.8
Sewage under the cages	37	27	73.0
Aisle floors	21	14	66.7
Breeder’s clothes	32	22	68.8
Equipment	26	15	57.7
Total	293	220	75.1

## 4. Discussion

For MiCV, there is no cell culture system for virus isolation and identification, and there is no effective vaccine or specific drug to prevent and control the disease. Some farmers used homemade inactivated vaccines (tissue supernatants of diseased mink inactivated using formalin) to prevent the disease and reduce morbidity; however, after a period of application, some minks developed symptoms of Aleutian disease. There are serious safety concerns with this type of vaccine; therefore, it has been discontinued (Wang et al., 2021). In view of this situation, the effective way to prevent the disease is to detect and eliminate the positive animals gradually in China. The recombinase polymerase amplification method had a minimum detection limit of 10 copies, but requires purification of amplification products and agarose gel electrophoresis to analyze the results (Ge et al., 2018c). The SYBR Green-based real-time PCR detection method for MiCV established by Cui could detect a minimum of 10^1^ copies/μl, in which the specific primers referred to the Cap gene sequences of eight different MiCV strains (Cui et al., 2018).

In this study, a quantitative real-time PCR technology based on TaqMan was established. The standard curve of the qPCR was established as shown in Figure 1. The correlation coefficient (R^2^) was 0.996 and the amplification efficiency was 93%, which indicated that the method established in this study is highly efficient, and mighty be a suitable approach for MiCV diagnosis. The primers and probes were designed according to the whole genome sequences of MiCV obtained in the laboratory in recent years and all 13 of MiCV genome sequences deposited in GenBank. Besides, six viruses that could cause potential cross-reactivity in qPCR assays were assessed. The qPCR method could successfully detect MiCV and without any cross reaction with other viral pathogens, including AMDV, MEV, CDV, CAdV-2, PRV, and PCV-2, indicating the high specificity and reliability of this method for MiCV detection. For the plasmid DNA, the minimum detection limit of the established qPCR method was 1.0 × 10^1^ copies/μl, whereas that for conventional PCR was 1.0 × 10^3^ copies/μl. For the virus DNA of MiCV, the minimum detection limit of the established qPCR method was 2.38 × 10^−2^ pg/μl, the conventional PCR was 2.38 × 10^1^ pg/μl. The results showed that the sensitivity of qPCR was higher than that of conventional PCR to detect MiCV. Moreover, the minimum detection limit of the qPCR method established in this study was lower than that of the previously established conventional PCR method and qPCR method (Wang et al., 2021). The values of the intra-assay CV ranged from 0.46 to 0.96%, and the values of the inter-assay CV ranged from 1.37 to 1.98%, which indicated that the qPCR method is highly reproducible.

MiCV infective rates in minks are high in some fur-bearing animal farms in China (Cui et al., 2018; Wang et al., 2021). In this study, we also detected a high positive detection rate of MiCV in minks, with positive detection rates exceeding 55% in Heilongjiang Province, Jilin Province, and Shandong Province. In clinical samples, the positive detection rate of MiCV in foxes was 52.4% in Heilongjiang Province, 50.9% in Jilin Province, and 48.9% in Shandong Province; in raccoon dogs it was 43.9% in Heilongjiang Province, 39.6% in Jilin Province, and 43.6% in Shandong Province. The total positive detection rate of MiCV in minks was higher (58.8%) than that in foxes (50.7%) and raccoon dogs (42.2%). However, the positive detection rates were lower than the 93% (40/43), 95.5% (21/22), and 69.2% (9/13) reported by Yang (Yang et al., 2018). We also found that the positive detection rate of MiCV in fur-bearing animals ≥ 1 year old was higher than that in animals <1 year old. The positive detection rate in male fur-bearing animals was lower than that in female fur-bearing animals. Usually when an infectious disease occurs on a farm, its pathogen can persist in the environment for a long time (Prieto et al., 2017). In this study we tested some environmental samples from cages, sinks, soil and sewage under cages. We found that the environmental samples of these fur-bearing animal farms have high positive detection rates. Among them, the samples from cages, troughs, soil under the cages, and sewage under cages had higher positive detection rates than the samples from aisle floors, breeder’s clothes and equipment. It is worth noting that these elements are frequently moved within the farm and in some exceptional cases are even transferred to other farms, which poses a significant risk to biosecurity. As far as we know, these results are the first application of the qPCR method to detect MiCV infection in environmental samples from fur-bearing animal farms. Unlike bacteria, there is no accepted standard culture method to quantify viruses in environmental samples; therefore, qPCR detection is a useful tool to study the epidemiology of viral diseases (Rodríguez-Lázaro et al., 2012). Although the presence of viral DNA cannot be interpreted as “infection” because its detection cannot determine infectivity, it might increase the risk of reinfection or transmission of the disease in the farms. These results focus attention on the role of environmental contamination in farms in the maintenance and transmission of this disease.

## 5. Conclusion

In this study, a highly sensitive, specific, repeatable and quantitative real-time PCR method for MiCV DNA detection was developed. The results showed that the positive detection rate of MiCV in minks was higher than that in foxes and raccoon dogs. The samples taken from the environment of the fur-bearing animal farms had high positive rates, and the positive detection rates in samples from cages, troughs, soil under the cages, and sewage under the cages were higher than those in the samples from the aisle floor, breeder clothes, and equipment. This study could contribute to control the spread of MiCV disease.

## Data availability statement

The original contributions presented in the study are included in the article/supplementary material, further inquiries can be directed to the corresponding authors.

## Ethics statement

The animal study was reviewed and approved by Animal Welffare and Research Ethics Committee of Jinlin Agricultural University (JLAU08201409).

## Author contributions

YL and CS: conceived of the study, carried out the experiment and drafted the manuscript, conducting a research and investigation process, specifically performing the experiments, or data/evidence collection YZ and JL: provision of study materials, reagents, materials, patients, laboratory samples, animals, instrumentation, computing resources, or other analysis tools. QG, KS, and FL: management and coordination responsibility for the research activity and execution, including mentorship external to the core team. LX and ZC: application of statistical, mathematical, computational, or other formal techniques to analyze or synthesize study data, preparation, creation and/or presentation of the published work, specifically visualization/data presentation. XL: conceived of the study and revising the manuscript critically. RD: acquisition of the financial support for the project leading to this publication. All authors contributed to the article and approved the submitted version.

## Funding

This research was supported by the Science and Technology Development Project of Jilin Province (grant numbers 20220101332JC and YDZJ202301ZYTS334) and the Science and Technology Research Project of Jilin Provincial Department of Education (grant number JJKH20210366KJ).

## Conflict of interest

The authors declare that the research was conducted in the absence of any commercial or financial relationships that could be construed as a potential conflict of interest.

## Publisher’s note

All claims expressed in this article are solely those of the authors and do not necessarily represent those of their affiliated organizations, or those of the publisher, the editors and the reviewers. Any product that may be evaluated in this article, or claim that may be made by its manufacturer, is not guaranteed or endorsed by the publisher.
